# Hydrogen Production Using TiO_2_-Based Photocatalysts: A Comprehensive Review

**DOI:** 10.1021/acsomega.3c00963

**Published:** 2023-07-14

**Authors:** Muhammad Rafique, Syeda Hajra, Muneeb Irshad, Muhammad Usman, Muhammad Imran, Mohammad A. Assiri, Waqar Muhammad Ashraf

**Affiliations:** † Department of Physics, University of Sahiwal, Sahiwal, Punjab 57000, Pakistan; ‡ Department of Physics, Faculty of Science, University of Gujrat, Gujrat, Punjab 50700, Pakistan; § Department of Physics, University of Engineering and Technology, Lahore, Punjab 54890, Pakistan; ∥ Department of Mechanical Engineering, University of Engineering and Technology, Lahore, Punjab 54890, Pakistan; ⊥ Research Centre for Advanced Materials Science (RCAMS), King Khalid University, P.O. Box 9004, Abha 61514, Saudi Arabia; # Department of Chemistry, Faculty of Science, King Khalid University, P.O. Box 9004, Abha 61413, Saudi Arabia; ∇ The Sargent Centre for Process Systems Engineering, Department of Chemical Engineering, University College London, London WC1E 6BT, U.K.

## Abstract

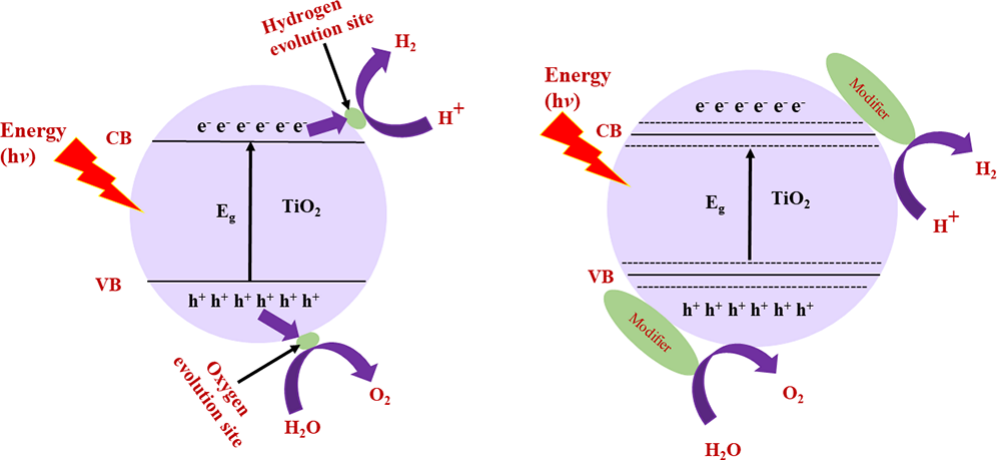

Titanium dioxide (TiO_2_) is one of the most widely used photocatalysts due to its physical and chemical properties. In this study, hydrogen energy production using TiO_2_- and titanate-based photocatalysts is discussed along with the pros and cons. The mechanism of the photocatalysis has been elaborated to pinpoint the photocatalyst for better performance. The chief characteristics and limitations of the TiO_2_ photocatalysts have been assessed. Further, TiO_2_-based photocatalysts modified with a transition metal, transition metal oxide, noble metal, graphitic carbon nitride, graphene, etc. have been reviewed. This study will provide a basic understanding to beginners and detailed knowledge to experts in the field to optimize the TiO_2_-based photocatalysts for hydrogen production.

## 1 Introduction

Hydrogen is considered an ideal fuel and is highly preferred due to properties such as its life cycle, renewability, environmental friendliness, and cost-effectiveness. Hydrogen can be produced from both renewable and nonrenewable energy resources. There are two main sources of renewable energy: solar energy and wind energy. These two sources are very suitable for the production of clean hydrogen. The main problem associated with these renewable resources is that only 5% of hydrogen is derived from them and they also involve high cost. While about 95% of hydrogen can be produced from nonrenewable resources, scientists are focusing on the methods through which they can produce cost-effective hydrogen. Then, the concept of photovoltaic water electrolysis was developed in which semiconductor materials that have small band gaps are used. This technology produced hydrogen at low costs. Alternatively, for hydrogen production, photocatalytic water splitting using TiO_2_ as a photocatalyst through solar energy is very promising. This way of producing hydrogen was very clean, low-cost, and environmentally friendly.^1−5^

**Figure 1:**
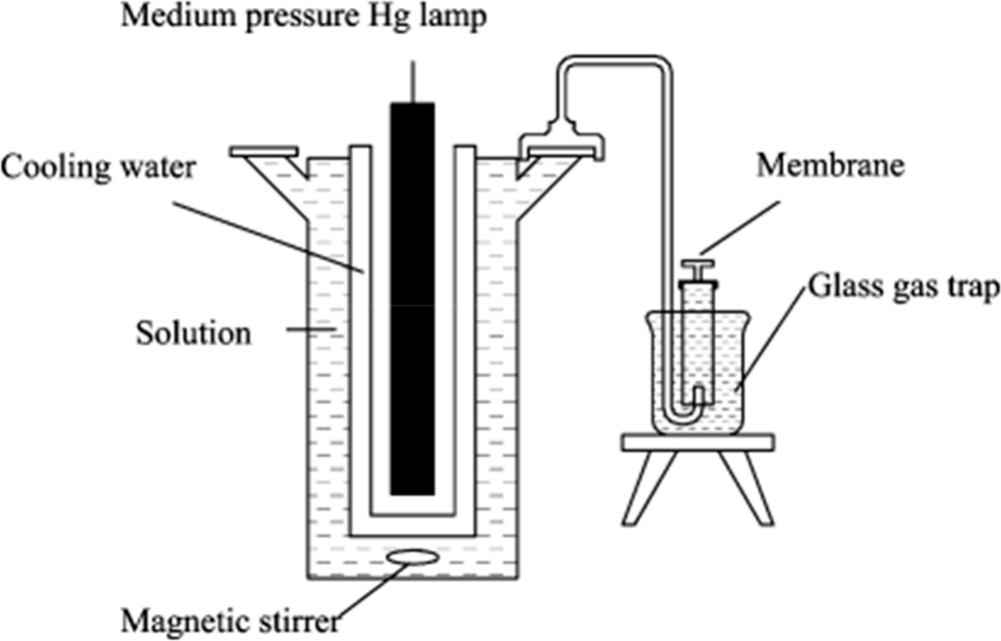
Apparatus for photocatalytic water splitting. Reprinted with permission from ref (6). Copyright 2005 American Chemical Society.

Catalytic hydrogen production using semiconductor materials as catalysts has attracted much attention because of the maximum utilization of solar energy. The apparatus for photocatalytic water splitting is shown in Figure 1. Photocatalytic reactions occur when semiconductor materials absorb photons with energy hυ equal to or greater than the band gap of the semiconductor. By absorbing this energy, electrons promote from the valence band to the conduction band and create an electron–hole pair. These photogenerated electrons promoted to conduction band and reduce H^+^ into H_2_, and holes on the semiconductor surface decompose H_2_O into O_2_ and H^+^.^6^ The behavior of these photogenerated carriers can have a significant influence on the performance of a semiconductor photocatalyst. Understanding and controlling the behavior of these carriers can lead to the evolution of efficient photocatalysts having a wide range of applications in environmental and energy-related fields. For example, the photogenerated charge carrier recombination can limit the photocatalyst efficiency. Similarly, controlling the transfer and migration of generated carriers can increase the efficiency of photocatalysts, such as the spatial separation of carriers, elongating their lifetime and thus increasing the photocatalytic performance. This behavior is explained in the following sections.

The overall mechanism of water splitting and hydrogen production is explained in eqs 1–4.^6−8^
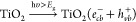
1
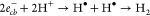
2
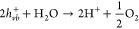
3

The overall reaction can be summarized as
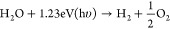
4

Titanium(IV) oxide naturally exists in two phases, namely, rutile and anatase, with a tetrahedral shape and simple synthesis.^5^ Meanwhile, the other third phase is called brookite, which can be synthesized in laboratories and is rhombic in shape. The photocatalytic activity of TiO_2_ such as anatase and rutile is influenced by crystal structure, surface area, surface hydroxyl density, porosity, and size^4,5,9^ because all these factors affect the production of electrons and holes. These two forms have mostly been used in photocatalytic applications. Anatase is one of the most active phases. The enhancement of photocatalytic activity is related to the Fermi level of about 0.1 eV in the anatase phase, which was higher than that in the rutile phase.^9,10^ TiO_2_ is available commercially called Degussa P25, which is used in photo catalytic studies. The hydrolysis of TiCl_4_ in hot flame produces TiO_2_ with a surface area of about 50 m^2^/g, and it contains a 4:1 ratio of anatase and rutile phases.^11^

### 1.1 Catalytic Mechanism

In this process, induced charge carriers cause the oxidation of electrons (such as donor species) in the valence band (VB) and the reduction of electrons (such as acceptor species) in the conduction band (CB). In the photomodernizing reaction, organic substrates behaved as electron donors while H^+^ was used as an electron acceptor. In this reaction, radiation energy is converted into chemical energy because in the presence of photocatalyst it absorbs solar energy very efficiently. In recent years, many different metal-oxide-based semiconductors as catalysts have been reported.^12^ Some metal oxides such as SrTiO_3_, TiO_2_, BaTi_4_O_9,_ ZrO_2_, and CeO_2_ have a reasonable ability to split water H_2_ and O_2_ under visible and ultraviolet light radiation.^13,14^ From all of these, TiO_2_ is favorable due to having a band gap of 3.2 eV in the anatase phase, high stability in the form of an aqueous solution under UV radiation, high reducing and oxidizing power, nontoxicity, and cost effectiveness.^3,5^

### 1.2 Limitations

There are some limitations while using TiO_2_, and the main problem facing TiO_2_ is its fast and undesirable electron–hole recombination reaction. In order to avoid this problem, sacrificial reagents can be used along with TiO_2_, which is also suitable to increase photo-efficiency. The main task of these sacrificial reagents is to keep separate the photoexcited electrons and holes from the recombination process or reversible process. Compounds such as methanol, EDTA (an ethylene diamine tetra acetic derivative), Na_2_SO_4_, ethanol, and Na_2_S and ions such as I^–^, IO^–^_3_, CN^–^, and Fe^3+^ are used as sacrificial reagents.^6^ Simply, we can say that the photocatalytic hydrogen efficiency obtained by simple TiO_2_ is low due to the following reasons: rapid recombination of photogenerated charge carriers, fast reverse reaction between H_2_ and O_2_, and large production of hydrogen, which becomes over potential.

Furthermore, in a simple aqueous system, pure TiO_2_ cannot split into H_2_ and O_2_. Therefore, to reduce these problems, many efforts have been made in recent years, such as the addition of sacrificial reagents, metal cation doping, carbon and nitrogen doping, and deposition of noble metals.^15,16^

Therefore, diverse techniques have been employed for the modification of TiO_2_ nanoparticles in order to attain the maximum possible hydrogen production rate. These methods involve the doping of transition metals, incorporation with other metal oxides, and surface modifications. Doping of transition metals in TiO_2_ surpasses the band gap of TiO_2_ by creating the quasi-static energy levels of dopants between the conduction and valence bands and also decreases the recombination rate of charge carriers.^17^ The reduction in the band gap of the TiO_2_ photocatalyst permits the material to harvest more photons during the reaction and thus produces more charge carriers. However, the incorporation of a metal oxide with TiO_2_ enhances its activity by transferring the photogenerated electrons to the conduction band in a lower position on the semiconductor while transferring holes to the less anodic valence band under the illumination of both semiconductors. This separation of charge carriers implies the reduction in the recombination rate and hence increases the photocatalytic performance.^18^ Therefore, it is claimed that TiO_2_ -based photocatalysts enhance the photocatalytic activity due to various factors such as an enhanced charge separation rate, a lower recombination rate, and the presence of oxygen vacancies.

As an example, a study affirmed the efficient hydrogen production rate of 23.5 mmol/g·h having an apparent quantum yield of 19% using the Ag-doped TiO_2_ photocatalyst. The observed efficient activity was attributed to the appearance of oxygen vacancies that enhanced the charge separation rate of (TiO_2_) responsible for the higher hydrogen production rate.^19^ Furthermore, a Ce3O4@C/TiO2 nanocomposite was fabricated by utilizing an incipient wet impregnation method for the photocatalytic production of hydrogen. The fabricated TiO_2_-based photocatalyst exhibited a hydrogen production rate of 11 400 μmol/g·h.^20^ These studies claimed the enhancement of the photocatalytic activity of TiO_2_ after its modification with different materials.

However, more fabricated modified TiO_2_-based photocatalysts are described in the proceeding sections.

## 2 Transition-Metal-Doped TiO_2_ Photocatalysts

### 2.1 Platinum (Pt)/TiO_2_ Photocatalyst

**Figure 2:**
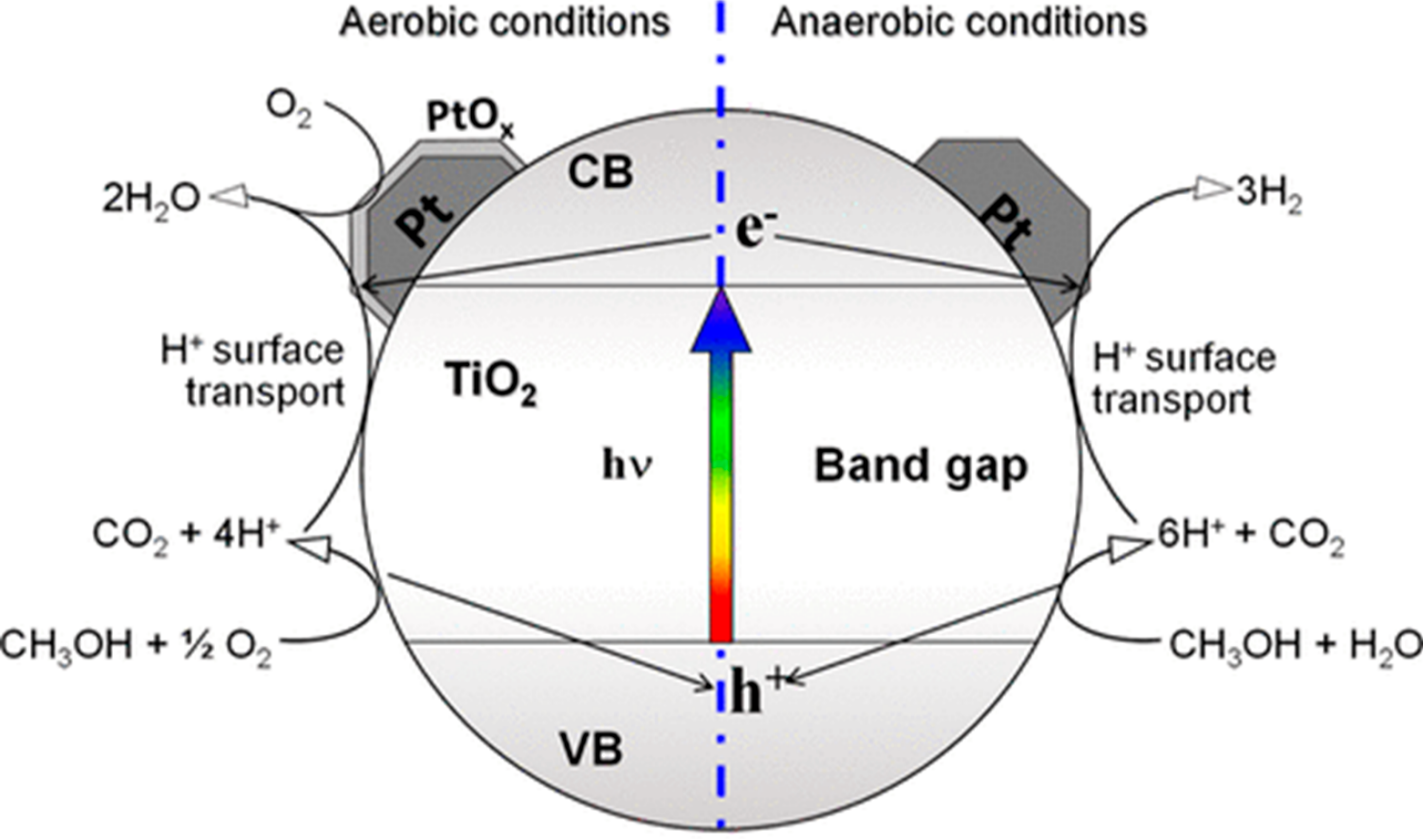
Mechanism of photocatalytic oxidation under aerobic and anaerobic conditions. Reprinted with permission from ref (22). Copyright 2022 American Chemical Society.

For the preparation of TiO_2_ nanosheets, a hydrothermal method was used. TiO_2_ nanosheets with exposed (001) faces were prepared in a mixed solution of Ti (OC_4_ H_9_)_4_–HF–H_2_O. Then, the deposition of Pt nanoparticles on the TiO_2_ nanosheets was carried out by a photochemical reduction method under xenon lamp radiation, and a Pt/TiO_2_ nanophotocatalyst is shown in Figure 2. The prepared sample was characterized by different characterization techniques such as scanning electron microscopy (SEM), photoluminescence spectroscopy (PS), X-ray diffraction (XRD), etc. Using coumarin as a probe material in photoluminescence (PL) spectroscopy, radicals of hydroxyl (OH•) were detected on the surface of the TiO_2_ nanosheets. However, the excited electrons, after migrating toward the conduction band of Pt nanoparticles, react with H^+^ ions to form the H_2_ molecule. In addition, the rates of photocatalytic H_2_ production were studied and discussed when Pt was loaded on the TiO_2_ nanosheets in an ethanol aqueous solution. It was shown by results that the loaded Pt on the TiO_2_ nanosheets enhanced the photocatalytic hydrogen production rates, and even 2 wt % deposited Pt showed the highest catalytic activity. Consequently, it was shown that, as compared to pure TiO_2,_ fluorinated TiO_2_ nanosheets exhibited high photocatalytic activity. These Pt/TiO_2_ nanosheets have attracted too much interest in different fields such as solar cells, sensors, biomedical engineering, photonic devices, and catalysis.^21,22^

The drawback of using Pt is that it is a rare and very expensive metal, so it has to be replaced, for which numerous efforts have been made.^15^ Moreover, when methanol was decomposed during the photocatalytic process on the Pt/TiO_2_ catalyst, carbon monoxide (CO), hydrogen, methane, and carbon dioxide were obtained. A concentration of about 2.7 vol % CO in hydrogen was observed. The concentration of CO in H_2_ was the main problem because a very small concentration of CO poisons the catalyst. In order to produce high amounts of and ultrapure hydrogen and for the reduction of the concentration of CO in H_2,_ scientists are focusing on some other catalysts that could be more suitable and cost-effective as compared to Pt/TiO_2_.^6,23^

### 2.2 Gold (Au) /TiO_2_ Photocatalyst

**Figure 3:**
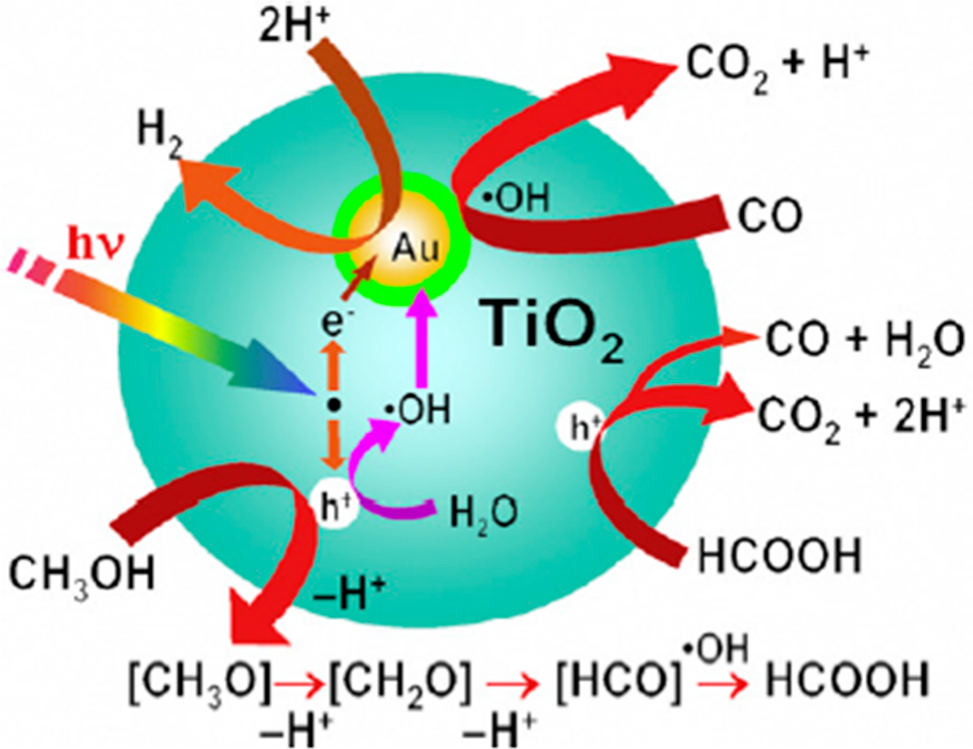
Schematic diagram of photocatalytic reaction on a Au/TiO2 catalyst. Reprinted with permission from ref (23). Copyright 2008 Elsevier.

For the production of H_2_, gold as a dopant over a TiO_2_ semiconductor can be selected as a catalyst in photocatalysis. The rate of H_2_ production is higher using a Au/TiO_2_ catalyst, as shown in Figure 3. H_2_ was produced during the photocatalytic decomposition of methanol on the Au/TiO_2_ catalyst using an ultralow concentration of CO. It was observed that when the size of the gold particles was decreased from 10 nm to smaller than 3 nm, the rate of H_2_ production was significantly increased. Additionally, it was perceived that the absorption of CO decreased when the size of the Au particles decreased. The suggested reason for reducing the concentration of CO was that when intermediate formic acid species form methanol through decomposition, mainly CO is produced. Because the size of the gold particles was reduced, the decomposition of formic acid (HCOOH) was stopped, which overturned the CO, and only H_2_ and CO_2_ were produced as byproducts.^6,23^

Bamwenda and co-workers made an experiment in order to compare the catalytic activity of Au/TiO_2_ and Pt/TiO_2_ catalysts for H_2_ production. TiO_2_ powder in aqueous suspensions of H_2_PtCl_6_·6H_2_O or HAuCl_4_·4H_2_O was used for the deposition of Pt and Au particles through a chemical deposition method. The byproducts obtained during this reaction were acetaldehyde, carbon dioxide, hydrogen, and formic acid. It was revealed the performance activity of the Pt sample was 30% higher than that of the Au sample. This is because of Au, which is highly dependent on preparation method as compared to Pt. When both Au and Pt samples was calcined at 573 K in air, they showed high production of hydrogen at this temperature. However, it was observed that the production of hydrogen become lower when calcination temperature was increased from 573 K temperature.^24^

### 2.3 Copper (Cu)/TiO_2_ Photocatalyst

**Figure 4:**
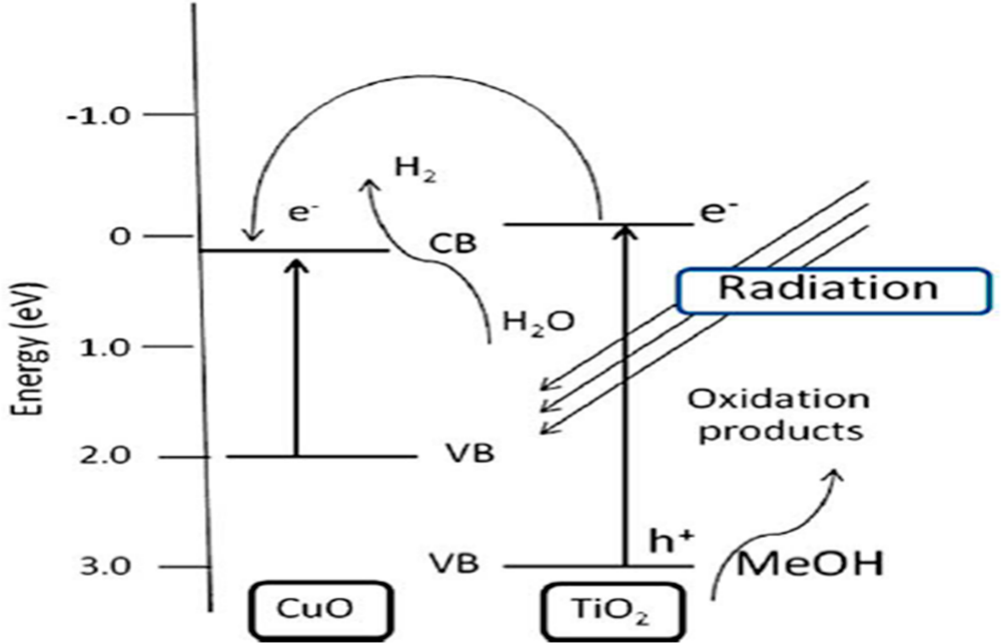
Schematic diagram of transfer and separation of charges of the Cu/TiO2 photocatalyst. Reprinted with permission from ref (25). Copyright 2009 Elsevier.

The photocatalyst of a Cu-doped TiO_2_ semiconductor material was used for the production of H_2_ under visible light. Complex precipitation and wet impregnation methods were used to prepare the Cu/TiO_2_ photocatalyst. The schematic diagram of the Cu/TiO_2_ photocatalyst is shown in Figure 4. The copper nitrate trihydrate was used as a starting material. The content of the copper dopant on the TiO_2_ semiconductor varied from 2% to 15%. The sample of the synthesized Cu/TiO_2_ photocatalyst was characterized by different characterization techniques, such as XRD, thermogravimetric analysis (TGA), SEM, and Fourier transform infrared spectroscopy (FITR). As mentioned above, the Cu/TiO_2_ photocatalyst was prepared by two synthesis methods under different concentrations of dopant and different calcination temperatures and showed different trends. The sample with 10% copper loading on TiO_2_ and a calcination temperature of 300 °C that was prepared by the complex precipitation method showed better photocatalytic activity than the other, which was prepared by the wet impregnation method. The size of the copper dopant particles varied from 20 to 110 nm. Its rate of H_2_ production was much higher than that of TiO_2_.^25^

### 2.4 Iron (Fe)- and Chromium (Cr)-Doped TiO_2_ Photocatalyst

Transition metals like Cr, V, Fe, Cu, Ni, and Mn are mainly used as dopants of titania in order to enhance the photoelectrochemical and optical properties of TiO_2_.^26^ As compared to other transition metals, Mn, Cu, and Fe can be used for trapping both charge carriers, namely, holes and electrons. While on the other hand, Ni and Cr can trap only one type of charge carrier.^1,27^ Two methods were used to synthesize the Fe- and Cr-doped TiO_2_ photocatalysts, namely, sol gel and radio frequency magnetron sputtering. It was observed that the Fe-doped TiO_2_ catalyst has a high rate of hydrogen production (such as 15.5 μmol/h) as compared to the Cr-doped TiO_2_ catalyst (only 5.3 μmol/h). This is because of the trapping ability of Fe, which can trap both types of charge carriers and also avoid the recombination process. However, Cr only traps one type of carrier and also causes the recombination process, which lowers its ability to produce more hydrogen as compared to Fe.^28^

## 3 Transition-Metal-Oxide-Doped TiO_2_ Photocatalysts

### 3.1 Indium Tin Oxide/Cr-Doped TiO_2_ Photocatalyst

To enhance the hydrogen production rate of a Cr-doped TiO_2_ catalyst, a single bilayer of indium tin oxide (ITO) is used with a Cr-doped TiO_2_ catalyst. Negligible photocurrent was observed due to increased recombination of charge carriers. To reduce this recombination process, multiple bilayers of indium tin oxide were deposited over TiO_2_. Then, it was observed that the photocurrent increased as the number of bilayers increased. The rate of hydrogen production obtained was about 24.5 μmol/h, which was two times greater than that of the pure titania (12.5 μmol/h).^29^

### 3.2 Cadmium Selenide (CdS)/TiO_2_ and Cobalt Oxide (CoO)/CdS/TiO_2_ Photocatalysts

**Figure 5:**
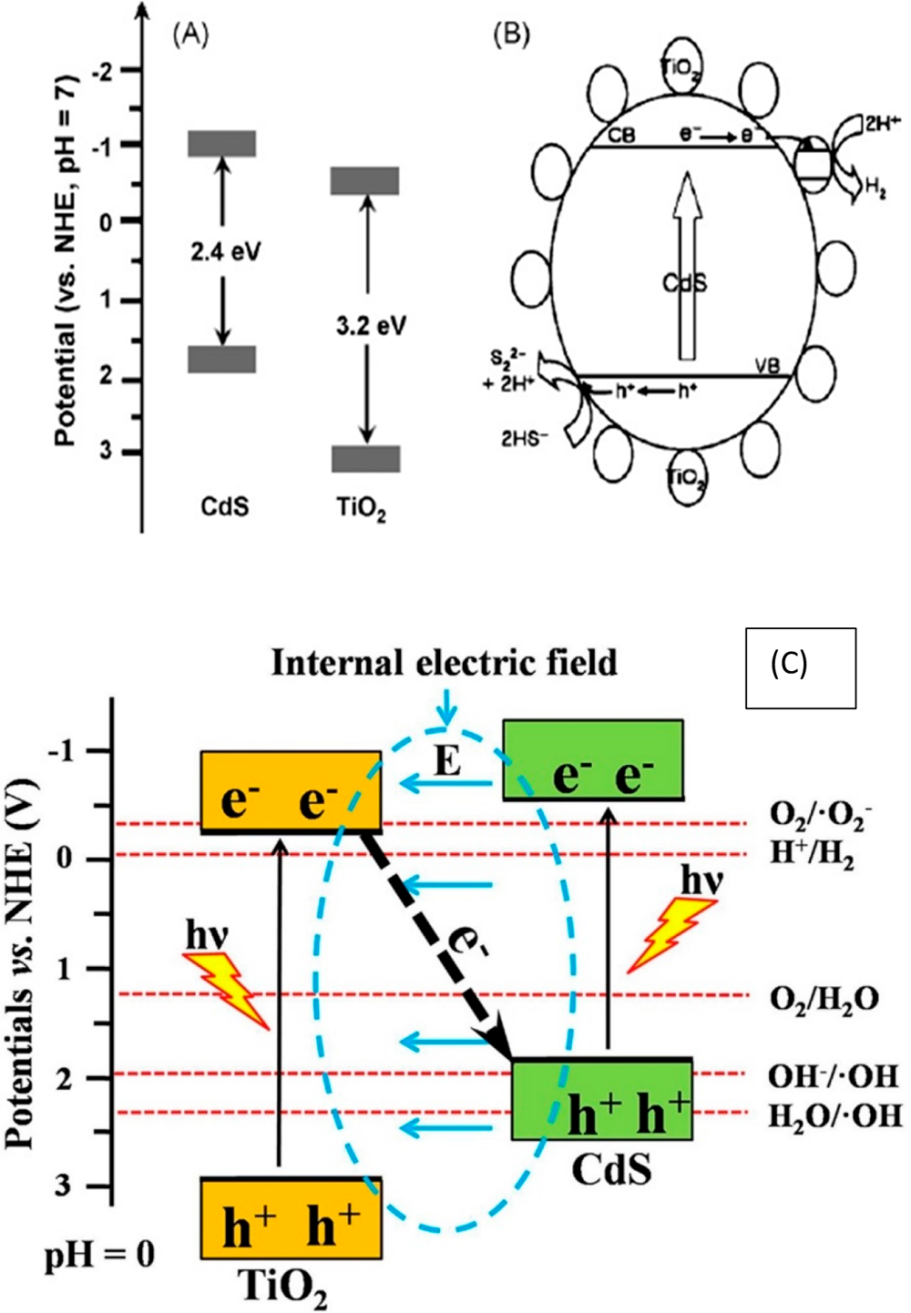
(a–c). Schematic diagram of the CdS/TiO_2_ photocatalyst. Panels a and b reprinted with permission from ref (30). Copyright 2007 Elsevier. Panel c reprinted with permission from ref (31). Copyright 2017 Elsevier.

CdS/TiO_2_ bulk nanocomposite material can be used as a photocatalyst. It was synthesized by using two methods, namely, sol gel and precipitation methods. A high H_2_ production rate was observed for the CdS/TiO_2_ photocatalyst, as shown in Figure 5. However,, CdS has some limitations, such as being unstable against photocorrosion. To overcome this problem, sacrificial agents are used in solutions such as S^2–^ and SO_3_^2–^. In the presence of these sacrificial agents, CdS showed high H_2_ production activity under light irradiation.^30^

**Figure 6:**
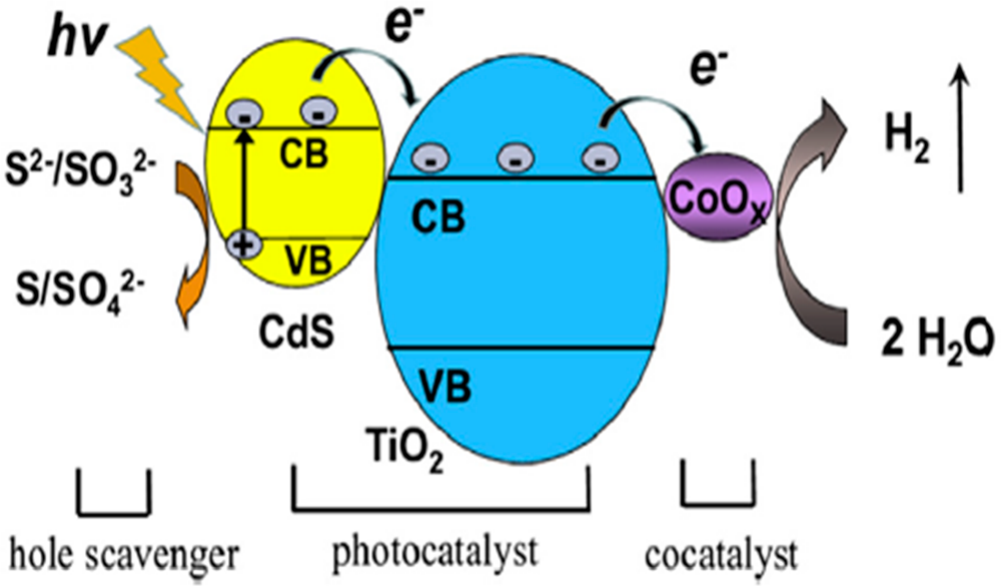
Schematic diagram of the CoO-loaded TiO_2_/CdS photocatalyst. Reprinted with permission from ref (32). Copyright 2014 Elsevier.

Recently, it was found that noble metal cobalt oxide (CoO) has been used as an electrocatalyst for proton reduction, and also it can be used as a photocatalyst for H_2_ production. CoO over TiO_2_/CdS was synthesized using a solvothermal method, as shown in Figure 6. These samples were characterized by TEM, XRD, and XPS in an aqueous solution (having sodium sulfite and sodium sulfide) as hole scavengers under visible light (λ > 400 nm). For a concentration of 2.1 wt % CoO, the hydrogen production rate was 660 μmol/g·h, which was seven times greater than that of the simple TiO_2_/CdS photocatalyst under the same conditions.^32^

### 3.3 Fe_2_O_3_/TiO_2_ Photocatalyst

**Figure 7:**
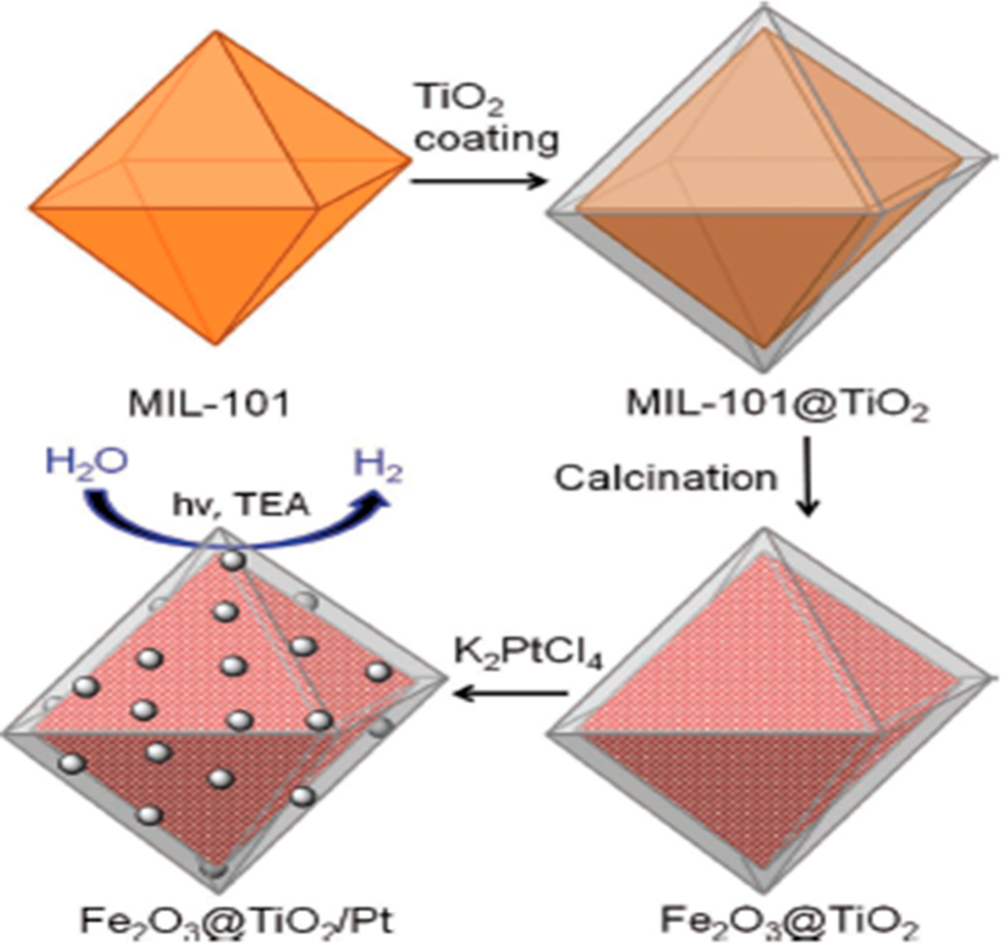
MOF template synthesis of Fe_2_O_3_/TiO_2_. Reprinted with permission from ref (33). Copyright 2012 John Wiley and Sons.

Metal oxide nanomaterials play a vital role in photocatalytic reactions to excite the electron to the CB and create a hole in the VB for the evolution of H_2_. These materials are interesting due to having high stability, low toxicity, and low-cost materials. A metal oxide such as TiO_2_ has a large band gap, which is suitable for photocatalytic fuel production but only absorbs UV radiation. For this reason, only 5% of energy is shown in the solar energy spectrum. Metal oxide nanomaterials such as Fe_2_O_3_ have suitable band gaps and are ineffective during photocatalytic reactions. A metal–organic framework (MOF) template is used to prepare the titania-based nanocomposite materials, as shown in Figure 7. Iron (Fe)-based MOFs are coated with titanium dioxide. Then, it was calcined to produce nanoparticles of composite Fe_2_O_3_/TiO_2_. This enables the composite to produce hydrogen when exposed to visible light radiation.^33^

### 3.4 Ni(OH)_2_/TiO_2_ Photocatalyst

**Figure 8:**
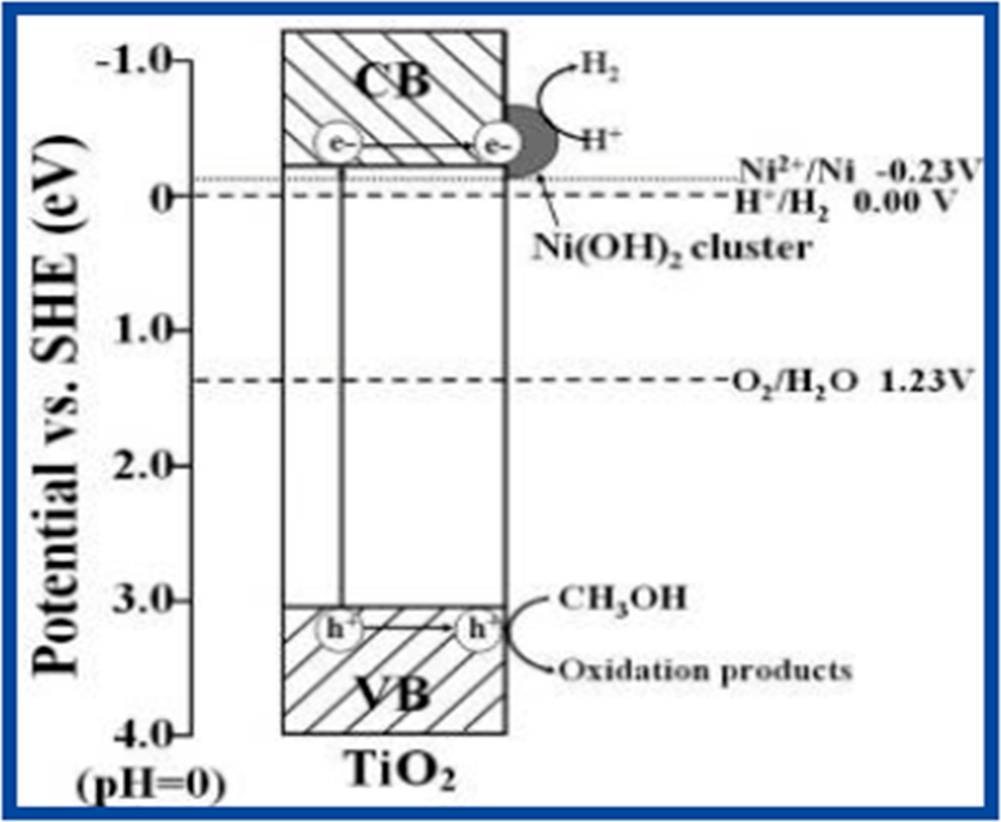
Schematic diagram of the transfer and separation of charges of the Ni(OH)_2_ cluster over modified TiO_2_. Reprinted with permission from ref (34). Copyright 2011 American Chemical Society.

A Ni(OH)_2_ cluster over TiO_2_ was used to synthesize a nanocomposite catalyst (Ni(OH)_2_/TiO_2_) via the precipitation method. In this process, Ni(NO_3_)_2_ was used as the precursor while the supportive material was Degussa P25 TiO_2_, as shown in Figure 8. It was observed that the rate of H_2_ production increased when a cluster of Ni(OH)_2_ was used within an aqueous solution of methanol. It was revealed that applying a Ni(OH)_2_ cluster on TiO_2_ increased the photocatalytic activity. Moreover, when 0.23 mol % Ni(OH)_2_ cluster was used, the rate of H_2_ production was increased by 3056 μmol/g·h. It was observed that the prepared sample had a 223× greater quantum efficiency as compared to that of pure TiO_2,_ which was obtained to be 12.4%. The main function of Ni^0^ is to separate charges and also for the reduction of water. The potential of Ni^2+^/Ni is more negative as compared to the H^+^/H_2_ potential, and it was also smaller than the CB of TiO_2_.^34^

## 4 Noble-Metal-Modified TiO_2_ Photocatalyst

TiO_2_ photocatalysts based on noble metals, such as Au, a Au alloy, and Pt, and Ag, can be synthesized by different methods, such as spray pyrolysis or deposition. When noble metals were deposited on TiO_2_, a good photocatalytic activity was observed. Moreover, by feeding methanol and water vapor on them, different byproducts were obtained, such as formaldehyde, carbon dioxide, methane, formic acid, dimethyl, methyl formate, ether, and acetaldehyde. These noble-metal-modified photocatalysts showed the best photocatalytic performance and produced a high H_2_ rate and low emission of CO. It was observed that Pt was best for a cocatalyst for the evolution of H_2_ as compared to other noble metals.^12^

## 5 Graphitic Carbon Nitride (g-C_3_N_4_)/TiO_2_ Photocatalyst

**Figure 9:**
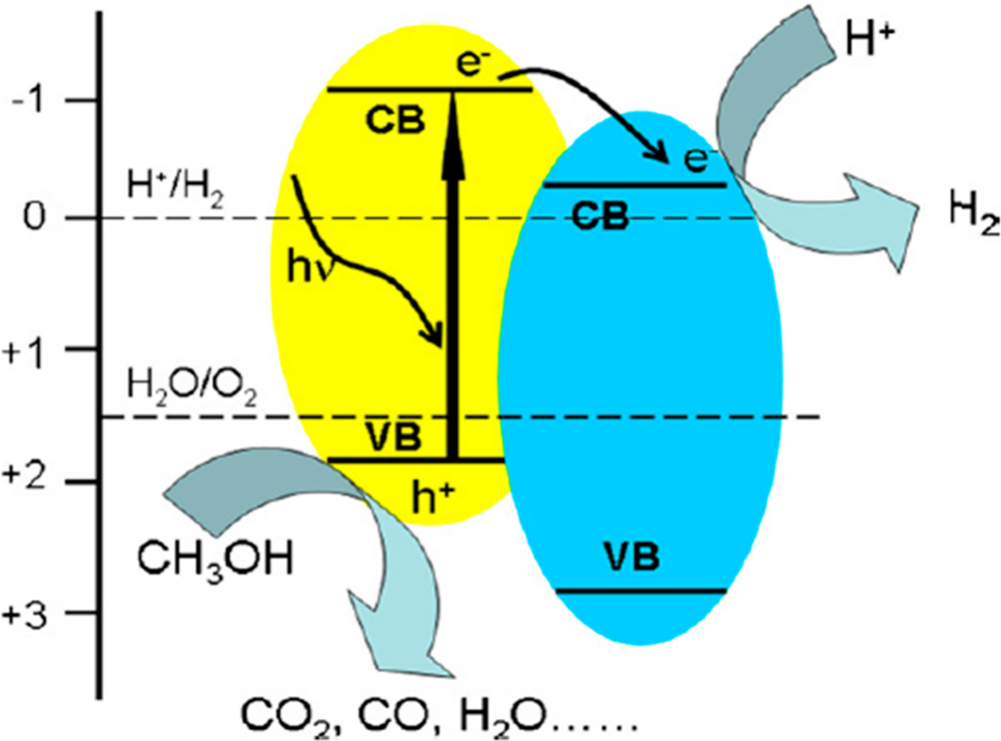
Schematic diagram of the transfer and separation of charges of composite TiO_2_ and g-C_3_N_4_. Reprinted with permission from ref (35). Copyright 2011 Elsevier.

Another polymer semiconductor, namely, g-C_3_N_4_ over TiO_2_, can be used as a photocatalyst in photocatalytic water splitting under visible light (in the presence of sacrificial reagents), as shown in Figure 9. The benefit of using this polymer semiconductor material was its optical band gap, which is 2.7 eV. Moreover, due to strong covalent bonds between nitride and carbon atoms, it shows high stability in water, acid, and base solutions under light irradiation. However,, the catalytic activity of this composite TiO_2_-g-C_3_N_4_ is reasonably acceptable, meaning very low.^35,36^

The composite g-C_3_N_4_/TiO_2_ catalyst was prepared and characterized by XRD and FTIR. It was shown that this composite material consisted of peaks of g-C_3_N_4_ and TiO_2_. The FTIR spectrum showed that this composite material has a stronger absorbance band intensity as compared to C_3_N_4_ alone. The hydrogen production rate under visible light was remarkably increased by coupling TiO_2_ with g-C_3_N_4_.^35^

### 5.1 g-C_3_ N_4_/SrTiO_3_-Based Photocatalyst

A g-C_3_N_4_-loaded SrTiO_3_ photocatalyst was synthesized by the decomposition of urea at 400 °C. g-C_3_N_4_ is a molecular photocatalyst in nature and has many advantages, such as a small band gap, facile absorption of visible light, and a high negative conduction band of about −1.12 eV, which causes facile transfer of photoelectrons to other components and is prepared by a simple and cheap method. In contrast, SrTiO_3_ has a conduction band level about −0.5 eV and a forbidden band gap about 3.2 eV; this property provides a close interfacial area to combine the g-C_3_N_4_ and SrTiO_3_. The rate of H_2_ production was observed to be nearly 440 μmol/g·h, which was larger than that of the simple anion-doped SrTiO_3_ photocatalyst.^37^

## 6 Graphene-Based GO/TiO_2_ Photocatalyst

**Figure 10:**
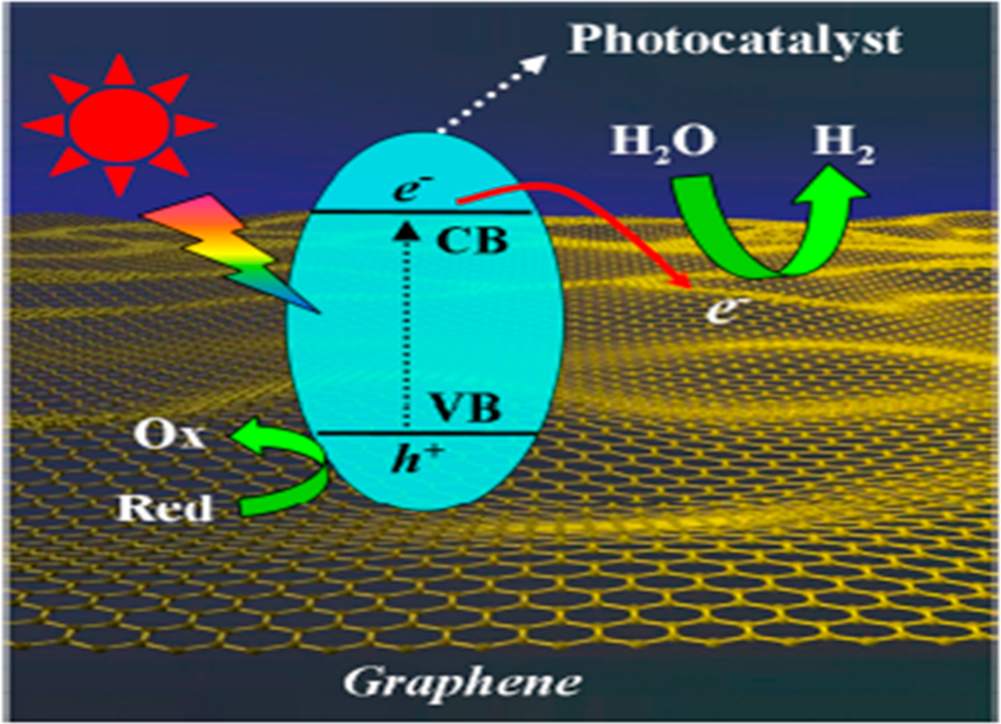
Proposed mechanism of graphene photocatalyst to enhance the photocatalytic performance. Reprinted with permission from ref (39). Copyright 2013 American Chemical Society.

Many researchers have expressed interest in rational designs of graphene for the performance of photocatalysts. A graphene-based photocatalyst converts solar energy into chemical energy in order to increase the H_2_ production, as shown in Figure 10. Graphene has different properties such as sp^2^ hybridization; due to this, it shows high thermal conductivity, which is about 5000 W/m·k. Graphene also offers excellent mobility of about 200·000 cm^2^/v· at room temperature, and its surface area is about 2600 m^2^·g.^38^ For these properties, graphene becomes more interesting as a photocatalyst because it has the ability to increase the transfer and separation of charge carriers. It enhances the efficiency in the following terms: reduces the recombination of electrons and holes, tunes the band gap of a semiconductor material, provides support to adsorption and catalytic sites, and acts as cocatalyst for producing hydrogen.^39,40^

**Figure 11:**
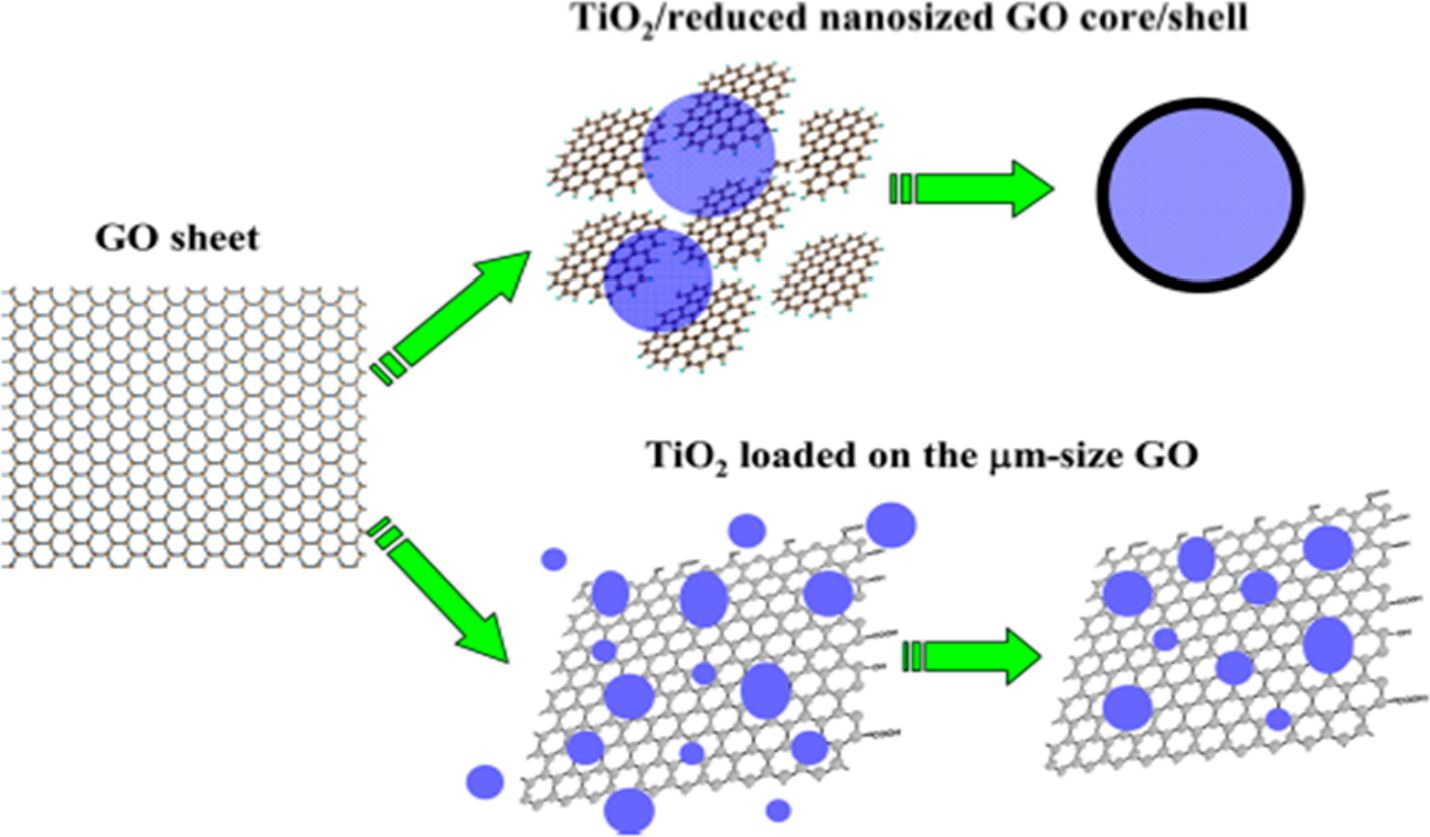
Schematic diagram of the preparation procedure of GO/TiO_2_ and TiO_2_/GO. Reprinted with permission from ref (39). Copyright 2013 American Chemical Society.

To increase the surface area of the photocatalyst, graphene provides two dimensional (2D) support and enhances its electrical and redox properties.^41^ The main problem was observed in using graphene when photocatalyst nanoparticles were applied on graphene sheets, and it was shown that a very small number of particles have direct contact with the graphene sheet, which causes a delay in the transfer of electrons in the photocatalytic reaction and creates weak interaction. To provide the larger specific surface area and strong interaction, the new structure of graphene was found in the form of nanosized graphene oxide with titania (GO/TiO_2_), as shown in Figure 11. This new developed structure of graphene has a self-assembled core and shell structure that shows a high rate of production of H_2_.^39,42^

### 6.1 Reduced Graphene Oxide (RGO)/TiO_2_ Photocatalyst

**Figure 12:**
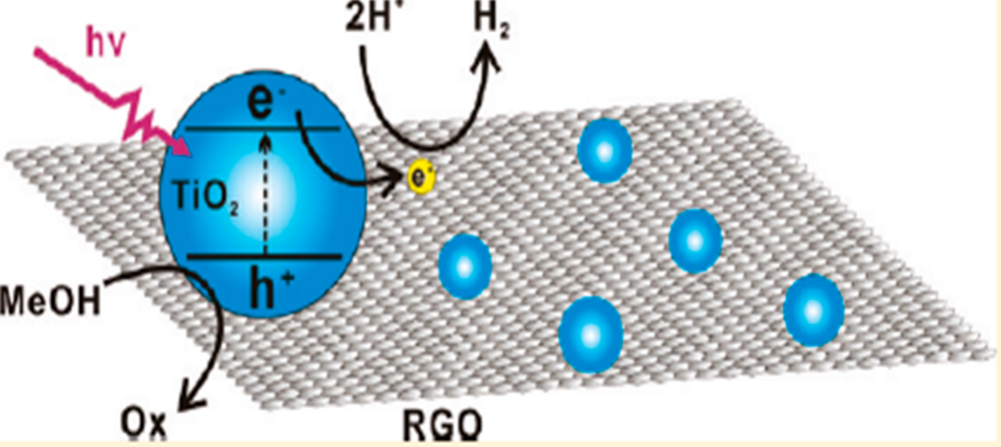
Schematic diagram of the RGO/TiO_2_ photocatalyst. Reprinted with permission from ref (43). Copyright 2011 American Chemical Society.

For the production of H_2_, RGO loaded over TiO_2_ can also be used as a photocatalyst in an alcohol solution under UV radiation, as shown in Figure 12. It was synthesized through the hydrothermal method, which showed the best photocatalytic activity and best performance. RGO/TiO_2_ contents in a ratio of 1:0.2 were used. The titanium dioxide (P25)–RGO was very stable and could be used as a recycle and catalyst for the evolution of H_2_. It was observed that when nanoparticles of P25 about 20–30 nm in size were loaded on RGO sheet, there was strong interaction between RGO and TiO_2_, which suppresses the recombination process and enhances the photocatalytic performance.^43^

There are few more recent studies reported that describe the peculiar behavior of modified TiO_2_ photocatalysts for the enhancement of hydrogen production rate. A study claimed the production of red phosphorus-modified TiO_2_ hollow spheres achieved the highest hydrogen production rate of about 215.5 μmol/g·h. It was revealed that the heterostructure incorporated photoinduced charge separation that enhanced the hydrogen production activity.^44^ Moreover, Bi/CdS/TiO_2_ nanocomposites were prepared via the successive ionic layer adsorption and reaction method. This nanocomposite manifested 673.81 μmol/h·cm^2^.^45^

Another study reported the preparation of a NiSe_2_ nanoparticle as a cocatalyst over TiO_2_ using a supercritical fluid process. The fabricated photocatalyst was investigated for hydrogen production and was revealed to possess a 219.2 mmol/g·h hydrogen production rate.^46^ Furthermore, a 2584.9 μmol/g· hydrogen production rate was revealed using the O-ZIS/TiO_2–*x*_ heterojunction.^47^

## 7 Conclusion

Hydrogen energy has become an emerging renewable energy resource because it is environmentally friendly, cost-effective, and has a stable life cycle. The most promising technique for hydrogen production is photocatalysis. The TiO_2_ photocatalyst is the most widely used in photocatalytic water splitting and hydrogen production. The noncommercial boundaries of the process are due to the catalyst limitation and are analyzed briefly in this Review. The process can be improved by doping and modifying the catalyst with transition metals, noble metals, graphite, and graphene and has been reviewed deliberately. This Review will provide insight into the TiO_2_ and TiO_2_-based photocatalysts to choose the best and optimized photocatalyst for water splitting and hydrogen production. There are various factors that must be focused on and considered to achieve the maximum hydrogen production activity, such as effective preparation methods, sacrificial reagents, photocatalyst stability and most importantly, hydrogen transport and storage as a future perspective.

However, it can be summarized that photocatalytic generation of hydrogen utilizing a TiO_2_ based photocatalyst is an economical and effective way to acquire sustainable energy. Doping of various nonmetal and metal materials can increase the performance of the photocatalyst by carefully tuning the band gap of the nanomaterials. Moreover, an optimum amount of the photocatalyst should be employed to achieve maximum activity because a high concentration of photocatalyst can reduce the efficiency of the photocatalyst.
